# Synthesis and characterization of CaSr-Metal Organic Frameworks for biodegradable orthopedic applications

**DOI:** 10.1038/s41598-019-49536-9

**Published:** 2019-09-10

**Authors:** Naomi Joseph, Harrison D. Lawson, Kalon J. Overholt, Krishnan Damodaran, Riccardo Gottardi, Abhinav P. Acharya, Steven R. Little

**Affiliations:** 1 Department of Chemical Engineering, University of Pittsburgh, Pittsburgh, PA 15261 USA; 2 Department of Bioengineering, University of Pittsburgh, Pittsburgh, PA 15261 USA; 3 Center for Cellular and Molecular Engineering, Department of Orthopaedic Surgery, University of Pittsburgh, Pittsburgh, PA 15261 USA; 4 Ri.MED Foundation, Palermo, Italy; 5 Division of Pulmonary Medicine, Department of Pediatrics, Children’s Hospital of Philadelphia, Philadelphia, USA; 6 Chemical Engineering, School for the Engineering of Matter, Transport, and Energy, Arizona State University, Phoenix, AZ 85287 USA; 7 Department of Pharmaceutical Sciences, University of Pittsburgh, Pittsburgh, PA 15261 USA; 8 Department of Ophthalmology, University of Pittsburgh, Pittsburgh, PA 15261 USA; 9 McGowan Institute for Regenerative Medicine, University of Pittsburgh, Pittsburgh, PA 15261 USA; 10 Department of Immunology, University of Pittsburgh School of Medicine, Pittsburgh, PA 15261 USA; 11 Department of Chemistry, University of Pittsburgh, Pittsburgh, PA 15261 USA

**Keywords:** Biomedical engineering, Regenerative medicine

## Abstract

Metal-organic frameworks (MOFs) formed from metals and organic ligands, are crystalline materials that are degradable in aqueous medium, and capable of releasing Ca and Sr ions. In this manuscript, the ability of MOFs to degrade and release osteogenic Ca and Sr ions was investigated. MOFs were generated by choosing osteoinductive Ca and Sr metals, and an organic ligand 1,3,5 tricarboxylicbenzene (H3BTC) as a linker. These MOFs were able to induce *in vitro* biomineralization from pre-osteoblastic MC3T3 cells and human mesenchymal stem cells (hMSCs). Moreover, these MOFs (when loaded with dimethyloxalylglycine (DMOG)) induced vascular endothelial production from hMSCs. qRT-PCR analysis performed on hMSCs (isolated from femoral heads of patients undergoing joint arthroplasty) treated with MOFs crystals suggested that the CaSr-MOFs by themselves can upregulate osteogenic genes in hMSCs, which is the first time to our knowledge that this has been observed from MOFs.

## Introduction

Hydroxyapatite remains the state-of-the-art material for osteogenesis because of its ability to provide a large reservoir of Ca for bone mineralization^1^. In addition to Ca, other metals such as Mg, Sr, Ce, Zn, Y and Al have been shown to have osteogenic properties^2,3^. Although, Mg based alloys have received the most interest, the degradation of bulk Mg in aqueous environments leads to the formation of hydrogen gas, which can be toxic^4^. In contrast, Ca and Sr are actively regulated in the body and both have been found to induce bone regeneration *in vivo*. Moreover, Sr, a natural trace element in human bone, can promote osteoblast growth and diminish bone resorption or breakdown^5^. Ca, also a natural ion within the body and bone, takes part in the formation of calcium phosphates during degradation to provide an optimal environment for local mineralization^6^. Additionally, alloys containing Ca and Sr were able to induce signaling pathways such as mitogen-activate protein kinases (MAPKs) which ultimately lead to osteoblastic differentiation^5^. The alloys were also able to upregulate several osteogenic genes such as *RUNX2*, *OSX*, and *ALP*^7^. Additionally, Zhang *et al*. and Berglund *et al*. demonstrated that the presence of Ca, Mg and Sr cations simultaneously can lead to bone regeneration^8,9^. Therefore, materials that can release Ca and Sr simultaneously for extended periods of time can provide osteogenic signals.

**Figure 1:**
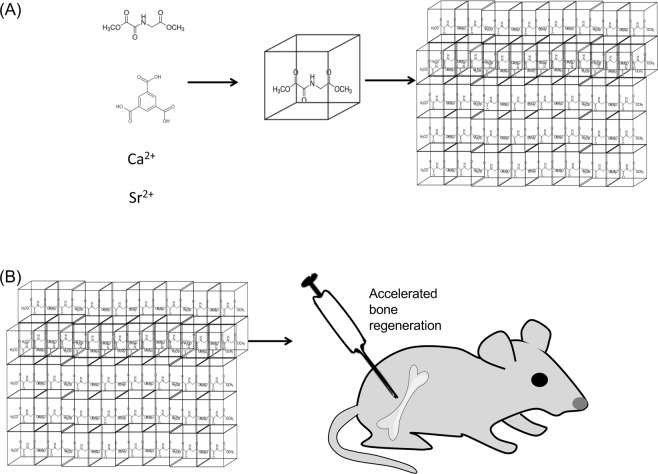
A schematic representation of the generation of CaSr-MOFs and their potential use in bone regeneration.

In this work, a new class of materials, metal organic frameworks (MOFs), were explored as a way to deliver Ca and Sr metals locally (Fig. 1). MOFs are frameworks of organic ions that coordinate with metal ions^10^, and are known to have the highest volume to mass ratio of any known material^11,12^. Given the high porosity and regular pore structure, MOFs can be loaded with drugs (such as small molecules drugs), which can then be released over time. In addition, the composition of a MOF has one half mole fraction of the entire crystal made up of metal ions, providing the potential to provide large reservoir of metal ions for bone mineralization. Overall, MOFs have the potential to be considered as coating for permanent implants, so that as the MOFs fully degrade in the body they will promote bone growth through controlled presentation of signals released from the framework and better implant integration.

To demonstrate proof in principle that MOFs could have beneficial osteogenic properties, we prepared a metal organic framework structure with trimesic acid (a tricarboxylic acid) as the organic ligand and Ca, and Sr as metal components. This structure was chosen given that dissolved Ca and Sr could provide osteogenic signals, and trimesic acid could aid in imparting rigidity to the porous MOF structure. Moreover, we were also able to show that a small molecule drug dimethyloxalylglycine (DMOG), which is a prolyl hydroxylase (PHD) pathway inhibitor and involved in bone regeneration, can be loaded into the MOFs. Using this new material, we performed a series of *in vitro* tests, including bone mineralization deposit assays, alkaline phosphatase production, and qRT-PCR-based analysis of osteogenic gene expression in pre-osteoblasts and human mesenchymal stem cells.

## Methods and Materials

### 1,3,5 tricarboxylic acid solution (H3BTC) preparation

Stock solutions of 100 mg/ml H3BTC were prepared by weighing 5 grams of H3BTC (Sigma Aldrich, St. Louis, MO) and added this amount to a re-sealable glass stock bottle. Then, 30 ml of Deionized (DI) water was added to the bottle. The contents were placed on a stirring plate and stirred at 650 rpm. Sodium Hydroxide 10 M (Fischer Scientific, Pittsburgh, PA) was added to the solution until all the H3BTC was dissolved. The solution was then titrated with 6 N HCl (Fischer Scientific, Pittsburgh, PA) until a pH range of 6.8–7.6 was achieved. DI water was then added until the desired concentration was achieved.

### MOF generation

Different cations (Ca^2+^, Sr^2+^, Mg^2+^) from chloride-hexahydrate salts (Fischer Scientific, Pittsburgh, PA), were weighed in combination (or separately) and added to a 20 ml scintillation vial. Different mole ratios of H3BTC and cations were prepared. The salts were dissolved in 4 ml of DI water and vortexed until completely dissolved. Then, 1 ml of H3BTC solution was added to the salt solution and gently stirred. This solution was then allowed to rest at room temperature for 3 days for MOF crystallization. After 3 days of incubation, MOFs were transferred into a 15 ml Falcon tube. The tube was spun down in a centrifuge at 2000 g for 5 min. The supernatant liquid was removed and 10 ml of water were added. The tube was again centrifuged. This processed was repeated until 3 washes were complete. The remaining supernatant was removed and the falcon tube with MOFs was placed in a lyophilizer until dry.

### DMOG MOFs

A stoichiometric amount of Ca^2+^ and Sr^2+^ were weighed out for a 1:1:1/2 (H3BTC: Ca: Sr) molar ratio solution and added to a 20 ml scintillation vial. Then 0.001, 0.01, 0.1 mole equivalent to H3BTC of dimethyloxalylglycine (DMOG) (Sigma Aldrich, St. Louis, MO) were added to the scintillation vial. The salts and DMOG were dissolved in 4 ml of DI water and shaken until completely dissolved. Then, 1 ml of H3BTC solution was added to the salt solution and gently stirred. This solution was then allowed to sit at room temperature for 3 days for MOF crystallization, washed and dried (as described in the previous paragraph).

### MOF characterization

MOF size and morphology were characterized using Scanning Electron Microscope (SEM JEOL JSM-6510LV/LGS), Electron Probe Micro Analyzer (EPMA JEOL JXA-8530F Scanning Electron Microscopy) and X-ray diffraction (XRD, High-Sensitivity Modular X-ray Diffraction System, Bruker D8 Discover with GADDS).

### Solid state carbon NMR

Solid-state Nuclear Magnetic Resonance (NMR) data was collected on a Bruker Avance Wide Bore 500 MHz spectrometer at ambient temperature using a 3.2 mm CP-MAS probe. ^1^H-^13^C Cross Polarization Magic Angle Spinning (CP-MAS) data was collected at multiple spinning rates to identify spinning side bands. For quantification purposes, ^13^C Direct Polarization (DP) with ^1^H decoupling data was collected at 15 kHz MAS using a 45° pulse and a recycle delay of 30 s. The ^13^C NMR chemical shifts were referenced to the carbonyl carbon resonance of labelled Glycine at 176.5 ppm.

### Degradation kinetics

Centrifuge tubes were filled with 10 mg of MOFs crystals. Phosphate Buffered Saline (PBS) solution without Mg and Ca (Fischer Scientific, Pittsburgh, PA) was added to the tube in 1 ml quantities. The tube was placed in the incubator at 37 degrees Celsius on a rotator. Every 24 hours, the samples were removed from incubation and spun down in the centrifuge at 1000 g for 5 min. Samples were drawn from the tube, approximately 800 µl of each sample, and placed in another Eppendorf tube for later testing. The removed PBS was replaced by fresh PBS and the tubes were placed back in the incubator on the rotator. The amount of Calcium ions collected was tested using a Quanti Chrom Calcium Assay Kit (BioAssay Systems, Hayward, CA).

### Cell cultures

MC3T3 cells (ATCC, Manassas, VA) were cultured in 10 ml of 10% fetal bovine serum (FBS) (FisherScientific, Pittsburgh, PA) and 1% penicillin/streptomycin (FisherScientific, Pittsburgh, PA) media. The cells were passaged every three days and no more than fifteen times. Then 20,000 cells were dispensed into 30 wells on a single 48-well plate. Each well also received 1 ml of phenol-free media and MOFs or control treatment. Pre-made MOFs were utilized to treat cells in 18 wells. Three types of MOFs were tested: Ca, Sr, and Ca-Sr. Initially Mg was also used as a MOF treatment however, it was decided to no longer utilize Mg because it did not aid in generating osteoblasts or proliferating cells. Of the remaining 12 wells, 6 were treated with 1 ml osteogenic media (100 nM of dexamethasone (FisherScientific, Pittsburgh, PA), 10 mM of glycerophosphate (FisherScientific, Pittsburgh, PA), and 50 µM of ascorbic acid (FisherScientific, Pittsburgh, PA)) to represent the positive control. The final 6 wells were not treated beyond 1 ml of phenol free media to indicate the negative control. On day 7, the sample underwent an MTT cell proliferation assay to ensure that the cells were metabolically active and alive after having been treated with MOFs. The samples were also simultaneously tested for bone differentiation via alkaline phosphatase assay after three and seven days.

### Isolation and expansion of hMSCs

All methods were carried out in accordance with relevant guidelines and regulations at University of Pittsburgh. All experiments were performed with cells from patients undergoing total joint arthroplasty with Institutional Review Board approval (University of Washington and University of Pittsburgh). Human MSCs were harvested from the femoral heads of patients undergoing joint arthroplasty and the cells were expanded as described by Lin *et al*.^8^. Cells were obtained from deidentified waste material from the operating room for which informed consent was not required as per Institutional Review Board approval. All experiments were performed with passage 3 (P3) MSCs from patients undergoing total joint arthroplasty with Institutional Review Board approval (University of Washington and University of Pittsburgh).

After expansion, MSCs were seeded in 6-well tissue culture plates at a density of 10,500 cells/cm^2^ and grown in basal medium (Dulbecco’s Modified Eagle Medium containing phenol red (FisherScientific), 10% FBS (FisherScientific) and 1% penicillin/streptomycin/fungizone (FisherScientific)). The cells were expanded until reaching 70% confluency, after which the medium was changed to osteogenic medium supplemented with either CaSr-MOFs or CaSr-DMOG-MOFs. Unsupplemented osteogenic medium was included as a control. To mitigate variation in gene expression due to donor age and sex, 3 pools of 3 donors each (9 donors total) were tested separately under the culture conditions outlined above. After 14 days, all cell monolayers were lysed using TRIzol for the extraction of RNA described below.

### MTT cell proliferation assay

In order to ensure that the cells survived and were proliferating, samples were treated with tetrazolium salts and reduced via dehydrogenase enzymes producing NADH or NADPH. Ten (10) µl of the yellow tetrazolium MTT (3-(4,5-dimethylthiazolyl-2)-2,5-diphenyltetrazolium bromide) (FisherScientific, Pittsburgh, PA) reagent was added to each sample well and then the cells were incubated for 2–4 hours until the purple precipitate became visible. Then 100 µl of dimethyl sulfoxide (DMSO) was added to each well and the cells were left at room temperature in the dark for 2 hours. Finally, the plate reader (Molecular Devices, Sunnyvale CA) was utilized to record absorbance of the dissolved crystals at 570 nm.

### Alkaline phosphatase assay

An alkaline phosphatase assay kit (Novus Biologicals, Littleton, CO) was utilized to test the levels of alkaline phosphatase in cell media. Sample Dilution Buffer was made by mixing 1 ml of the 10X dilution buffer with 9 ml of sterile water. Next, a 400 ng/ml SEAP standard stock solution was created by diluting 1 μl of SEAP protein with 499 μl of the sample dilution buffer in an Eppendorf tube. Finally, a 5 mM p-nitrophenyl phosphate (*p*NPP) solution was prepared by diluting 1 ml of the 10X *p*NPP buffer with 9 ml of sterile water and mixing in a 5 mg *p*NPP substrate tablet with 5 ml of the PNPP substrate solution.

This test was conducted for 21 days. In a reaction well of a 96-well plate, 50 μl of cell conditioned medium was dispensed. Then, 50 μl of 5 mM *p*NPP solution was added to the wells. A standard curve was also prepared by conducting a 1-to-2 dilution. In 8 wells 50 μl of the 1X Dilution Buffer was dispensed, and in the first well of the series, 50 μl of the 400 ng/ml SEAP standard was also dispensed and mixed in solution. Then from the first well, 50 µl of the solution was extracted and dispensed into the next well in the series. This process was repeated until that last well which only contained 50 μl of Dilution Buffer. The outputs were measured on a microplate reader at 405 nm wavelength after one hour of allowing the reaction to carry out in the absence of light.

### OsteoImage mineralization assay

OsteoImage (FisherScientific, Pittsburgh, PA) was conducted to determine the ability of MOFs to induce osteoblast cells to generate bone minerals. First, two solutions were initially prepared before the start of the assay: wash buffer and staining reagent. The 10X stock Wash Buffer was diluted to 1X with deionized water. The Staining Reagent was diluted 100-fold in Staining Reagent Dilution Buffer. The solution was mixed well and protected from light during the duration of the assay.

After 21 days the plates to be evaluated were removed from the incubator and cooled to room temperature. The media from each well was removed and the wells were washed once with PBS. Then the cells were fixed by incubating the cells with formalin (FisherScientific, Pittsburgh, PA) for 20 minutes. After fixing the cells, the wells were rinsed twice with 0.4 ml of 1X Wash Buffer. Next, 0.2 ml of diluted Staining Reagent was added to each well and then the plate was incubated at room temperature protected from the light for 30 minutes.

After 30 minutes, the Staining Reagent was removed from the wells and the wells were washed 3 times with 0.4 ml of diluted Wash Buffer. Each wash was left in the wells for approximately 5 minutes. After the last wash, 0.4 ml of Wash Buffer was added to each well and images were obtained using a fluorescent microscope using 492 nm excitation and 520 nm emission. Moreover, a plate reader (SpectraMax, Molecular Devices), was utilized to quantify the fluorescence from each well using 492 nm excitation and 520 nm emission.

### RNA isolation and cDNA synthesis

Human cell monolayers, cultured as described in *Isolation and Expansion of hMSCs*, were collected and suspended in TRIzol Reagent (Invitrogen, Waltham, MA, USA). Total RNA was isolated using a combination of liquid-liquid extraction and spin column approaches. Briefly, an RNA-rich aqueous layer was separated from the TRIzol suspension in an acid guanidium thiocyanate-phenol-chloroform extraction and the organic layer was discarded. The RNA-containing aqueous suspension was further purified using a Qiagen RNeasy Plus Mini Kit according to the manufacturer’s instructions (Qiagen, Germantown, MD, USA), and the total RNA concentration was measured with a Nanodrop 2000c Spectrophotometer (Thermo Fisher Scientific, Waltham, MA, USA). Single-stranded cDNA was synthesized using a SuperScript IV kit (Invitrogen) following the manufacturer’s protocol.

### Quantitative real-time polymerase chain reaction (qRT-PCR)

Quantitative real-time polymerase chain reaction (qRT-PCR) was performed using SYBR Green PowerUp Master Mix reagents (Applied Biosystems, Foster City, CA, USA) and oligonucleotide primers (Integrated DNA Technologies, Coralville, IA, USA). The expression of the following osteogenic genes was analyzed: alkaline phosphatase (*ALP*), bone sialoprotein 2 (*BSP II*), runt-related transcription factor 2 (*RUNX2*), osteocalcein (*OCN*), osteopontin (*OPN*), and collagen type I, alpha 1 (*COL1A1*, referred to as *COL1* throughout). 18S rRNA (*18S*) was analyzed as a stably expressed reference gene. The primer sequences and their locations in the human transcriptomic database are listed in Table 1. All qRT-PCR reactions were performed using a StepOne Plus real-time thermocycler (Applied Biosystems) and the fold changes in mRNA expression from baseline values were normalized to 18S expression levels and calculated using the comparative CT method (ΔΔCT).

**Table 1:** Primer sequences used for qRT-PCR, along with accession numbers in the NCBI nucleotide database.

Gene	Forward Primer Sequence (5′-3′)	Reverse Primer Sequence (5′-3′)	Product Size (bp)	Accession Number
*18S*	GTAACCCGTTGAACCCCATT	CCATCCAATCGGTAGTAGCG	151	NR_146146.1
*ALP*	ATCTTTGGTCTGGCCCCCATG	AGTCCACCATGGAGACATTCTCTC	124	NM_000478.6
*BSP II*	CGAATACACGGGCGTCAATG	GTAGCTGTACTCATCTTCATAGGC	109	NM_004967.4
*RUNX2*	CAACCACAGAACCACAAGTGC	TGTTTGATGCCATAGTCCCTC	196	NM_001024630.4
*OCN*	ATGAGAGCCCTCACACTCCTC	GCCGTAGAAGCGCCGATAGGC	294	NM_199173.6
*OPN*	TCACCAGTCTGATGAGTCTCACCATTC	TAGCATCAGGGTACTGGATGTCAGGT	203	NM_001040058.2
*COL1A1*	TAAAGGGTCACCGTGGCT	CGAACCACATTGGCATCA	355	NM_000088.3

### Statistical analysis

Statistical analysis was performed using GraphPad Prism 7. Changes in gene expression were analyzed via a one-way ANOVA test with repeated measures (using the Greenhouse-Geisser correction), followed by Tukey’s multiple comparisons post-hoc testing. Statistical significance was qualified using a threshold of p < 0.05. All data were expressed as mean ± standard deviation.

## Results and Discussion

**Figure 2:**
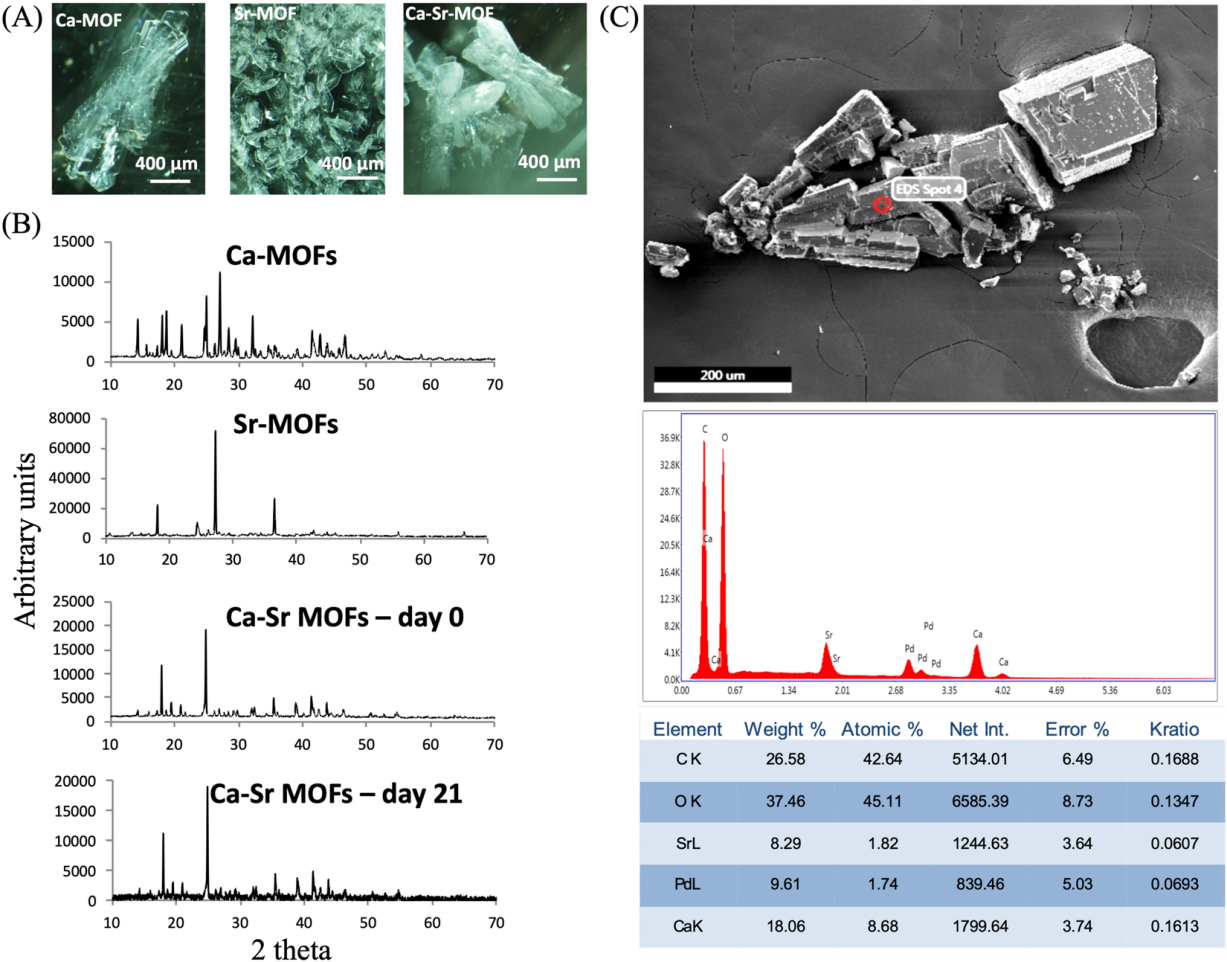
Characterization of CaSr-MOFs. (**A**) Optical images of Ca-MOF, Sr-MOF and CaSr-MOF. (**B**) XRD spectra of different synthesized crystals. (**C**) A representative EDS analysis of CaSr-MOFs.

In this study, MOF crystals were designed that could deliver both calcium (Ca) and strontium (Sr) ions as well as an encapsulated molecule. The 1:0.5 mole ratio of Ca:Sr has been reported to provide an osteogenic differentiation signal to stem cells^3^. Therefore, in this study Ca and Sr were mixed together at room temperature with trimesic acid (H3BTC) as the organic linker. Control crystals of single metal centers were also generated under the same reaction conditions (Supplementary Figs S1–S8). Room temperature synthesis was chosen as that would preserve the activity of drugs that could potentially be encapsulated in the future. The weight of incorporated Ca and Sr was assessed by energy-dispersive X-ray spectroscopy (EDS) (Fig. 2C) and confirmed a Ca to Sr ratio of approximately 1:0.5 was incorporated in the MOF structure. The crystals of H3BTC:Ca:Sr (Fig. 2A, Ca-H3BTC and Sr-H3BTC are shown as controls), were stable for >21 days (sharp peaks from XRD data suggested crystalline structure was maintained – Fig. 2B). Crystals were also generated with a 1:1:1:0.5 mole ratio of H3BTC:Ca:Mg:Sr and imaged by SEM (Fig. S8). The H3BTC:Ca:Mg:Sr crystals were several hundred microns in size, and uniform with well-defined edges. Unfortunately, when these crystals were re-suspended in water, they dissolved immediately, and could not therefore be used for the local sustained delivery of Ca and Sr to cells. Moreover, H3BTC:Ca:Sr crystals (hereby referred to as CaSr-MOFs) at about 100 microns in size were markedly smaller than H3BTC:Mg:Ca:Sr crystals, an overall desirable feature for local delivery systems processability. Calcium ion release from MOFs was also assessed (Supplementary Fig. S9) and after a brief burst in the first 2 days, it stabilized to a continuous, uniform release up to day 24.

**Figure 3:**
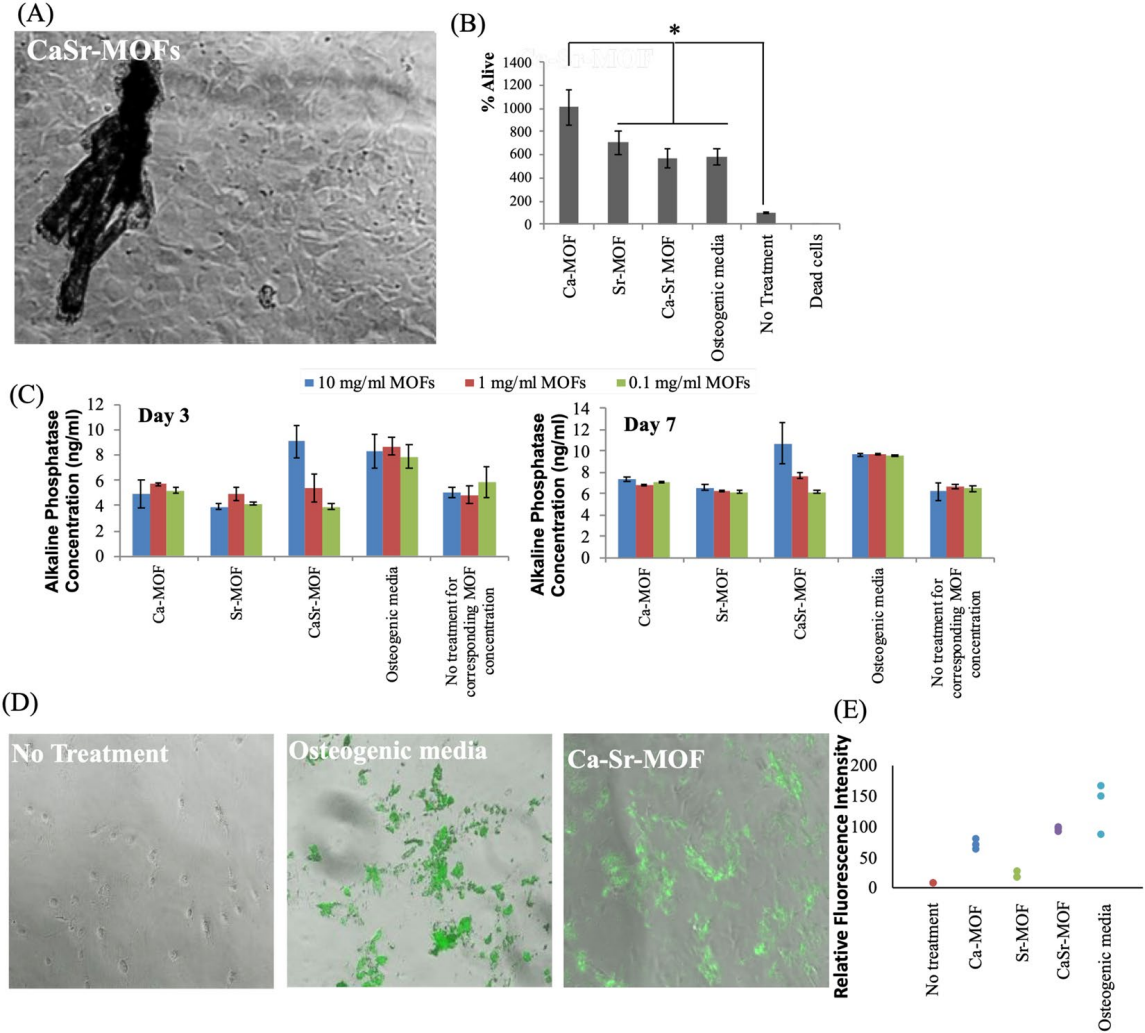
CaSr-MOFs induce differentiation of pre-osteoblastic cells to osteoblastic cells, and induce bone mineralization. (**A**) A brightfield image of CaSr-MOFs with MC3T3 cells. (**B**) Assay for determining %alive MC3T3 cells in the presence of different MOFs. (**C**) Alkaline phosphatase generation by MC3T3 in the presence of different MOFs for day 3 and day 7 culture. The no treatment group is the negative control for the corresponding concentration of MOFs. (**D**) Bone mineralization (shown in green) by MC3T3 in the presence of CaSr-MOF compared to positive (osteogenic medium) and negative (no treatment) controls. (**E**) Quantification of bone mineralization for treatment with different MOFs.

In order to investigate if the MC3T3 pre-osteoblastic cells proliferate in the presence of CaSr-MOFs, these cells were cultured in the presence of the MOF crystals for 7 days (Fig. 3A shows brightfield image of MOFs and MC3T3 cell co-culture) and cell metabolism was assessed by an MTT assay. Dead cells and no treatment were utilized as controls. Figure 3B shows that the Ca-MOFs induced the highest number of cells. Moreover, Sr-MOFs, CaSr-MOFs and osteogenic media were not significantly different from each other, but were significantly higher than the no treatment control. These data suggest that the MOFs generated here can lead to higher proliferation of MC3T3 cells or be associated with a marked increase in cell metabolism which is consistent with differentiation. This data is also supported by other reports that suggest that Ca and Sr can lead to proliferation of pre-osteoblastic cells^13,14^. This modulation of cell activity has been attributed to calcium-sensing receptor signaling, which induced several genes responsible for mineralization.

To determine if MOFs induce up-regulation of osteogenic markers, on day 3 and day 7 of culture with MOF treatment, alkaline phosphatase levels were assessed using a commercial alkaline phosphatase kit. Figure 3C illustrates the concentrations of alkaline phosphatase for each sample following treatment with three different concentrations of MOFs (0.5 mg/ml, 1 mg/ml, and 10 mg/ml). The CaSr-MOFs induced the highest alkaline phosphatase production from MC3T3 cells at 10 mg/ml concentration, which was not significantly different from the positive control of osteogenic media, and significantly higher than all other groups at both day 3 and day 7. Furthermore, MC3T3 cells were treated with 10 mg/ml of CaSr-MOFs and mineral deposition was assessed using the OsteoImage assay. Figure 3D shows representative images of the hydroxyapatite deposition (green) for CaSr-MOFs compared to negative (no treatment) and positive (osteogenic medium) controls and qualitatively appears almost as good as the positive control, as confirmed by the quantification of total hydroxyapatite deposition reported in Fig. 3E. Notably, CaSr-MOFs induced higher bone mineralization (hydroxyapatite) from MC3T3 pre-osteoblastic cells than individual metals (Ca-MOFs and Sr-MOFs). These data suggest that the CaSr-MOFs may release Ca and Sr from the matrix and with a cumulative effect on osteoinduction. These results are supported by previous studies performed with alloys of Mg-Ca-Sr, which suggested that Ca and Sr by themselves may lead to osteoinduction^8,9^.

**Figure 4:**
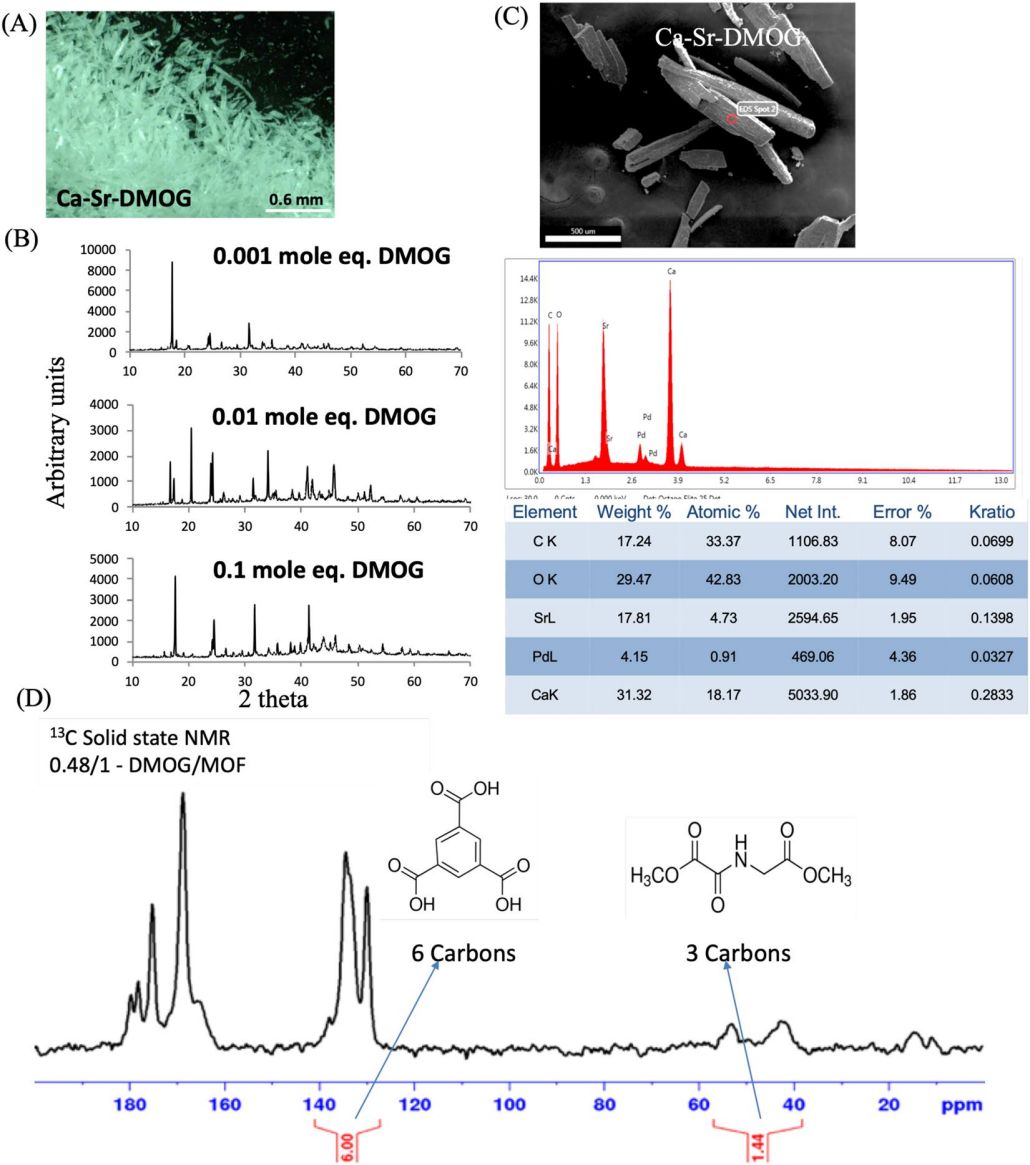
Characterization of DMOG incorporating CaSr MOFs. (**A**) Optical image of DMOG encapsulated CaSr-DMOG. (**B**) XRD analysis of DMOG encapsulated crystals. (**C**) EDS analysis of DMOG encapsulated MOFs. (**D**) ^13^C NMR of DMOG encapsulated MOFs.

In addition to providing osteogenic signals through biomaterials, bone-regeneration is also supported by providing access to nutrients via vascularization to the regenerative bone tissue. It has been suggested that delivery of the small molecule DMOG (inhibitor of Prolyl hydroxylase (PHD) pathway)^15^ can lead to increased vascularization, via vascular endothelial growth factor (VEGF) production from hMSCs^16,17^. Since the CaSr-MOFs described here have a large pore volume, they also have the potential to be utilized to load small molecule drugs like DMOG, which could induce higher expression of VEGF in the local environment. Correspondingly, we introduced DMOG during the synthesis of CaSr-MOFs to load it into the metal organic framework structure. Specifically, different concentrations of DMOG were added during the synthesis of the crystals (representative image of crystals - Fig. 4A). XRD analyses suggested that although the introduction of different quantities of DMOG during synthesis changed the intensity of the peaks, the overall crystal structure remained the same, and new peaks were not observed (Fig. 4B). Analyses revealed that a Ca:Sr ratio of approximately 1:0.5 was achieved in the MOF structure (Fig. 4C). Lastly, in order to investigate if DMOG was being encapsulated/associated with the crystals, ^13^C solid state NMR was performed. The carbon peak corresponding to DMOG and H3BTC were observed, and upon quantification provided the mole ratio of 0.48:1 of DMOG:H3BTC (Fig. 4D). These data suggest that CaSr-MOFs were able to encapsulate DMOG, at a loading that was significantly higher (>2-fold, mg/mg of loading in drug delivery material) than other delivery systems reported in the literature^18–20^.

**Figure 5:**
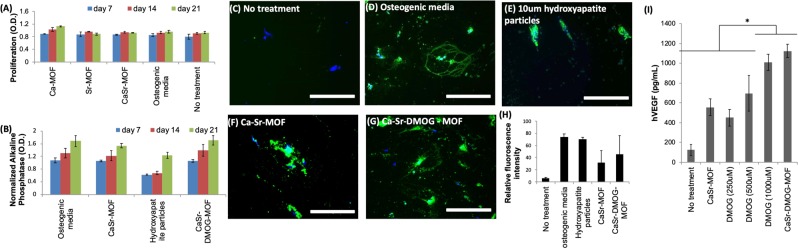
CaSr-MOFs induce bone mineralization from hMSCs. (**A**) Prolfieration of hMSCs in the presence of MOFs. (**B**) Normalized alkaline phosphatase production by hMSCs in the presence of different MOFs and hydroxyapatite particles. **(C–H)** Bone mineralization (shown in green) by hMSCs in the presence of different MOFs and hydroxyapatite particles at day 21, and its quantification is graphed. **(I)** hVEGF produced by hMSCs when cultured in the presence of different concentrations of DMOG and DMOG encapsulated Ca-Sr-MOFs.

In order to determine if DMOG loaded CaSr-MOFs can initiate VEGF production, ELISA was performed on the supernatant of hMSCs cultured with CaSr-DMOG-MOFs. It was observed that the MOFs were able to induce significantly higher levels of VEGF than a solution of 1000 μM soluble DMOG, with no treatment and CaSr-MOF alone as controls (Fig. 5I). These data suggest that CaSr-DMOG-MOFs might be able to release DMOG at the vicinity of the hMSCs and thus may support bone regeneration via induction of VEGF from these cells. The ability of Ca-Sr-DMOG MOFs to produce this effect *in vivo* will be the focus of future work.

We then tested the DMOG loaded MOFs for their ability to induce differentiation of hMSCs toward osteogenic lineage and produce *in vitro* bone mineralization. Accordingly, CaSr-DMOG-MOFs and CaSr-MOFs were compared with hydroxyapatite particles (lower porosity than MOFs, but widely used for bone mineralization) and soluble Ca for their capacity to induce bone mineralization in hMSCs. The hMSCs were cultured with 10 mg/ml of CaSr-MOFs or CaSr-DMOG-MOFs, soluble Ca (0.1 μmolar), 10 mg/mL of hydroxyapatite particles (10 μm in size), osteogenic media or without any treatment. These concentrations and culture conditions were not toxic to cells as shown in Fig. 5A. Furthermore, alkaline phosphatase levels (normalized to no treatment) increased similarly for all conditions at day 7, 14, and 21 (Fig. 5B). Finally, after 21 days of culture hydroxyapatite deposition was qualitatively (using fluorescent microscope) and quantitatively (fluorescence spectroscopy) determined using the OsteoImage assay. Although quantitatively the hydroxyapatite particles, osteoinductive media, CaSr-MOFs, and CaSr-DMOG-MOFs all generated similar amount of fluorescence, the fluorescence signal was distributed differentially in these conditions (Fig. 5C–H). Specifically, the fluorescence in the condition with hydroxyapatite particles was localized closely around the particles themselves, whereas the surface area of induced mineralization was larger in the MOFs conditions, similar to osteoinductive media. This suggests an effect of controlled release of the metal ions diffusing from the MOFs which would correspond to this increased area of induced mineralization.

**Figure 6:**
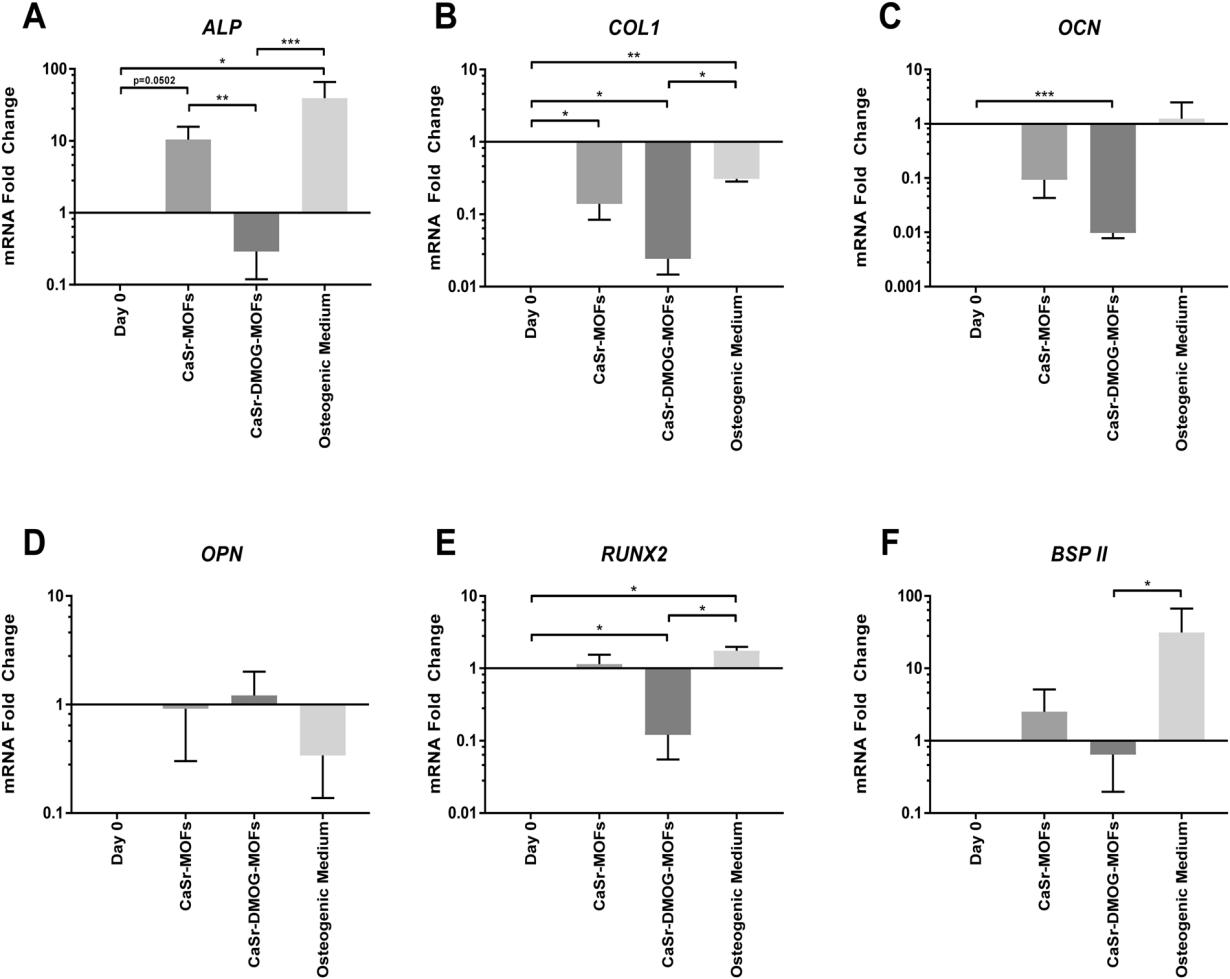
Relative expression levels of mRNA transcripts for osteogenic genes in hMSC monolayers after treatment with MOFs. Results are shown after 14 days of treatment with CaSr-MOFs, CaSr-DMOG-MOFs, or osteogenic medium. (**A**–**F**) Expression of *ALP, BSP II, RUNX2, OCN, OPN*, and *COL1*. Expression levels are graphed on a log_10_ scale as fold changes normalized to day 0 baseline values and then to 18S rRNA levels (*p < 0.05, **p < 0.01, ***p < 0.001).

In addition to investigating the functional outputs of CaSr-MOFs, we tested the MOFs ability to modulate osteoblast-specific mRNA levels of hMSCs. To this end, CaSr-MOFs and CaSr-DMOG-MOFs were applied to hMSC cultures isolated from the femoral heads of patients undergoing joint arthroplasty. The synthesis of osteoblast-specific mRNA transcripts was assessed by qRT-PCR after 21 day of culture to determine whether MOFs can induce hMSC differentiation towards an osteoblastic lineage. Specifically, modulation of *ALP, BSP II, RUNX2, OCN, OPN*, and *COL1* expression was investigated and the relative changes in gene expression are shown in Fig. 6A–F. It was observed that CaSr-MOFs were able to induce upregulation of the differentiation markers *ALP* and *BSP II* from initial levels, albeit non-significantly, with p = 0.0502 for *ALP* falling near the defined significance of p = 0.05. CaSr-MOFs also caused significant downregulation in *COL1* from day 0 (p = 0.0434). DMOG-containing CaSr-DMOG-MOFs produced significantly lower expression of *COL1*, *OCN*, and *RUNX2* compared to day 0 levels (p = 0.0124, 0.0002, and 0.0441 respectively). For the positive control osteogenic treatment, upregulation from day 0 of *ALP* and *RUNX2* was significant (p = 0.0257 and 0.0446 respectively), while increase in *OCN* and *BSP II* was not. Additionally, a significant downregulation in *COL1* occurred in the osteogenic control treatment (p = 0.0033). Comparing between treatment groups, the expression of *ALP* was significantly higher for CaSr-MOF treatment than for CaSr-DMOG-MOF treatment (p = 0.0046). Furthermore, treatment with CaSr-DMOG-MOFs resulted in significantly decreased expression of *ALP, COL1, RUNX2*, and *BSP II* compared to the control osteogenic treatment (p < 0.0001, p = 0.0346, 0.0187, and 0.0422 respectively). Gene expression analysis revealed that treatment of hMSCs with CaSr-MOFs upregulated *ALP* similarly to treatment with osteogenic medium, though osteogenic treatment produced a larger effect. Our observations of *ALP* gene expression match the analysis of alkaline phosphatase production shown in Fig. 4C: alkaline phosphatase production was slightly increased at 1.0 mg/mL of CaSr-MOFs, though not to the same extent as in the osteogenic medium control group. Notably, a much larger increase of alkaline phosphatase occurred with a CaSr-MOF concentration of 10.0 mg/mL, although this concentration was not tested by qRT-PCR. Conversely to the observations of *ALP* expression, the downregulation of *COL1* was more extreme for CaSr-MOFs than for osteogenic treatment. Though osteogenic media produced an upregulation of the early osteogenic gene *RUNX2*, this was not observed for CaSr-MOFs. Notably, all three treatments down-regulated *COL1* despite its usual function as an osteogenic gene. This deficit may be remediated by increasing the concentration of ascorbic acid in the media. Interestingly, treatment with CaSr-DMOG-MOFs significantly down-regulated the osteogenic genes *OCN* and *RUNX2* from their initial levels. Nonsignificant trends showed that DMOG-containing MOFs may also downregulate *ALP*, *COL1*, and *BSP II*. Comparing between treatment groups, it can be concluded that CaSr-DMOG-MOFs broadly oppose the trends of the control osteogenic treatment, with CaSr-MOFs producing an intermediate condition which remarkably more often parallels the expression pattern in osteogenic medium. Overall, these data suggest that CaSr-MOFs alone appear to provide osteogenic signals to hMSCs and promote osteogenic differentiation in the absence of any exogenous growth factor. However, the poor performance of the CaSr-DMOG-MOFs remains puzzling as others have previously demonstrated that DMOG can accelerate bone mineralization *in vivo*^15,21,22^, possibly via Rho/ROCK signaling^23^. Notably, our osteogenic media does not contain growth factors such as bone morphogenic protein (BMP) nor vitamin D3 which may act synergistically on some of the same pathways^24,25^. It is then possible that outcomes of CaSR-DMOG-MOFs exposure would be dramatically altered in different culture conditions or *in vivo* in the presence of endothelial cells. In fact, the cross talk between stem cells differentiating towards an osteogenic phenotype and vascular endothelial cells enhances both osteogenesis and vascularization^26^. It is then possible that *in vivo* the increased VEGF production in the MSCs induced by DMOG could enhance endothelial cell osteogenic feedback signaling. Furthermore, a key component of our culture media was dexamethasone, which possesses significant anti-inflammatory potential and supports pro-osteogenic differentiation. Recently, MOFs containing dexamethasone have been used to coat a titanium substrate showing good cytocompatibility and supporting osteogenic differentiation in a mouse cell line^27^. Reasonably, similar outcomes could be expected with human cells, and it would then be very promising to combine dexamethasone MOFs with CaSr-MOFs as coatings for titanium implants to promote osseointegration and ostoegenesis.

## Conclusion

In conclusion, this is the first study (to our knowledge) to investigate the ability of ion releasing MOFs to influence osteogenic activity in human cells. MOFs containing Ca and Sr were generated that were able to induce proliferation and differentiation of MC3T3 cells and hMSCs. Moreover, CaSr MOFs were able to encapsulate DMOG and induce VEGF production from hMSCs. Lastly, it was observed that CaSr-MOFs were able to upregulate osteogenic markers in hMSCs obtained from human donors.

## Supplementary information

Synthesis and characterization of CaSr-Metal Organic Frameworks for biodegradable orthopedic applications
