# Supplementary material for: Synthesis and characterization of CaSr-Metal Organic Frameworks for biodegradable orthopedic applications
Source: Sci Rep. 2019 Sep 10;9:13024. doi: 10.1038/s41598-019-49536-9 (PMC6736967; doi:10.1038/s41598-019-49536-9)
Supplement: Supplementary file 1 — Synthesis and characterization of CaSr-Metal Organic Frameworks for biodegradable orthopedic applications [file 41598_2019_49536_MOESM1_ESM.docx]

**Synthesis and characterization of CaSr-Metal Organic Frameworks for biodegradable orthopedic applications**

**Naomi Joseph*^1^, Harrison D. Lawson*^1^, Kalon J. Overholt^2^, Krishnan Damodaran^11^, Riccardo Gottardi^1,3,4,5,9^, Abhinav P. Acharya^#6^, Steven R. Little^#1,2,7,8,9,10,11^**

*1 Department of Chemical Engineering, University of Pittsburgh, PA, 15261*

*2 Department of Bioengineering, University of Pittsburgh, PA, 15261*

*3 Center for Cellular and Molecular Engineering, Department of Orthopaedic Surgery, University of Pittsburgh, PA, 15261*

*4 Ri.MED Foundation, Palermo, Italy*

*5 Division of Pulmonary Medicine, Department of Pediatrics, Children's Hospital of Philadelphia*

*6 Chemical Engineering, School for the Engineering of Matter, Transport, and Energy, Arizona State University, AZ, 85284*

*7 Department of Pharmaceutical Sciences, University of Pittsburgh, PA, 15261*

*8 Department of Ophthalmology, University of Pittsburgh, PA, 15261*

*9 McGowan Institute for Regenerative Medicine, University of Pittsburgh, PA, USA, 15261*

*10 Department of Immunology, University of Pittsburgh School of Medicine, PA, 15261*

*11 Department of Chemistry, University of Pittsburgh, PA, USA, 15261*

*** - Contributed equally**

**# - Corresponding authors**

**Correspondence to abhi.acharya@asu.edu, srlittle@pitt.edu**

**Supplementary Information and results:**

**Results of single metal centers with H3BTC.**

MOF formulations were generated using a 1:0.5 mole ratio mixture of H3BTC and Ca, and a control with no H3BTC at room temperature. Figure S1 represents the control vial that showed no crystal formation in the absence of H3BTC. Figure S2 shows SEM micrographs of the crystals obtained using the Ca mixture confirming successful production of crystals in the presence of H3BTC. The SEM images show 1:0.5 Ca crystals having a non-uniform and flakey or 2D structure. Crystal sizes varied around a few microns in length. Since the structure of these crystals were not uniform, further formulations were then tested. MOF formulations of 1:1 and 1:2 (H3BTC:Cation) were made with Ca, Sr, and Mg. The crystalline structure of each formulation was imaged using an optical microscope (Fig. S3-S5) and SEM (Fig. S6-S7). Considering crystal size and uniformity, the 1:1 molar ratio of cations to H3BTC produced the formulations with the most uniformity in crystal structure (Figures S7 and S8). Magnesium crystals were not imaged because they solvated after re-suspension in water. From Figures S6 and S7, it can be inferred that the H3BTC:Ca - 1:1 mole ratio formulation gave the largest and most uniform crystals. Moreover, H3BTC:Sr generated large and uniform crystals as compared to H3BTC:Ca, and H3BTC:Sr - 1:1 ratio developed fewer uniform crystals as compared to H3BTC:Sr - 1:2. These results demonstrated that crystals can be generated at room temperature using H3BTC, Ca and/or Sr.

**Results of calcium ion release from MOFs.**

In order to determine if the CaSr-MOFs can release Ca ions, ICP-MS was performed. Specifically, 10 mg of MOF crystals were incubated in 1 mL of water for 1 day at 37°C. The supernatant was removed and replaced with fresh 1 mL of water. This step was repeated every day for 24 days, and the amount of calcium ion in the supernatant was determined using a standard curve. It was observed that the MOFs were able to release Ca ions every day for 24 days.


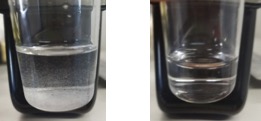


**Figure S1**. The presence of H3BTC (left figure) is required for the generation of crystals and no crystals are generated (right figure) in the absence of it. This suggest that metal salts are not precipitating to generate the crystals, and the crystals can be the co-ordination of H3BTC and the metal ions.

**
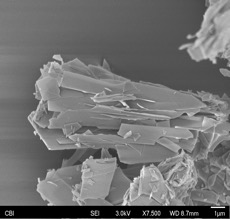
**

**Figure S2**. SEM images of the Calcium (1:1/2) crystals at x7,500, (Scale bar = 1 μm).


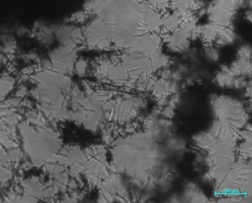

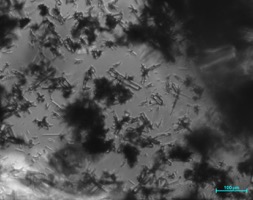


A

B

**Figure S3.** Microscope images (10x magnification) of the 1:1 (A) and 1:2 (B) Calcium: H3BTC crystals 3 hours after settling (Scale bar = 100 μm).


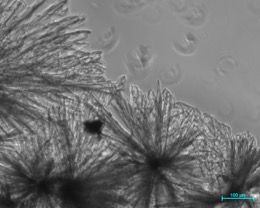

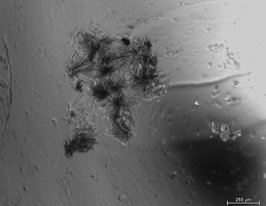


B

**Figure S4.** Microscope images of 1:1 (A), at 10x magnification, and 1:2 (B), at 10x magnification, Magnesium: H3BTC crystals 3 hours after mixing (Scale bar = 100 μm).


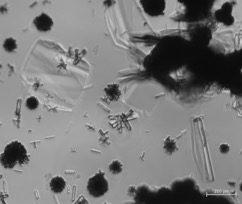

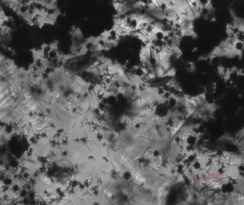


A

B

**Figure S5.** Microscope images (10x magnification) of the 1:1 (A) and 1:2 (B) Strontium: H3BTC crystals 3 hours after settling (Scale bar = 100 μm).


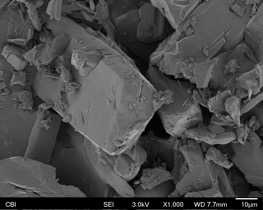

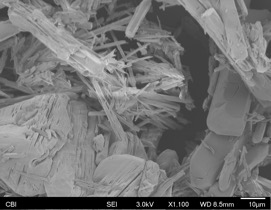


A

B

**Figure S6.** (A) 1:1 (Calcium: H3BTC) MOFs at x1000. (B) 1:2 (Calcium: H3BTC) MOFs at x1100, (Scale bar = 10 μm).


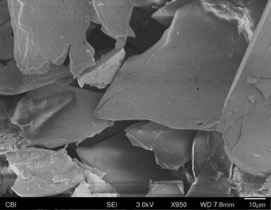


A

B


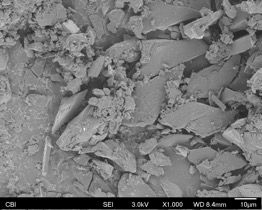


A

**Figure S7.** 1:1 (Strontium: H3BTC) MOFs at x1100. (B) 1:2 (Strontium: H3BTC) MOFs at x950, (Scale bar = 10 μm).


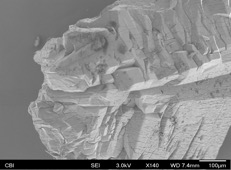


**Figure S8.** SEM images of 1:1:1/2 (Ca: Mg: Sr) and H3BTC MOFs, scale bar = 150 μm.

**Figure S9.** Release kinetics of calcium shows burst release for the first 3 days, and then slow continuous release till day 24.
